# Vertical Alignment of Liquid Crystals on Phenylphenoxymethyl-Substituted Polystyrene—PS Derivatives Structurally Similar to LC Molecules

**DOI:** 10.3390/polym14050934

**Published:** 2022-02-25

**Authors:** Jihyeon Moon, Chaewon Kang, Hyo Kang

**Affiliations:** BK-21 Four Graduate Program, Department of Chemical Engineering, Dong-A University, 37 Nakdong-Daero, 550 Beon-gil, Saha–gu, Busan 49315, Korea; 1829469@donga.ac.kr (J.M.); codnjs1120@gmail.com (C.K.)

**Keywords:** liquid crystal, precursor, alignment layer, polystyrene, 4-phenylphenol

## Abstract

A series of polystyrene derivatives containing precursors of liquid crystal (LC) molecules, phenylphenoxymethyl-substituted polystyrene (PPHE#; # = 5, 15, 25, 50, 75, and 100)—where # is the molar content of 4-phenylphenol using polymer modification reactions—were prepared in order to examine the effect of the polymer film, which possess similar LC molecular structure on the LC alignment properties. It was found that the *T*_g_ values of the PPHE# were higher than 100 °C due to their aromatic structure in the biphenyl-based PHE moiety. The LC cells fabricated with PPHE5 and PPHE15 films exhibited planar LC alignment. Conversely, LC molecules showed a vertical alignment in LC cells made using the polymer films with phenylphenoxymethyl side groups in the range of 25–100 mol %. The polar surface energies on the PPHE# films can be associated with the vertical LC alignment on the PPHE# films. For example, vertical LC alignment was exhibited when the polar surface energy of the polymer films was less than approximately 4.2 mJ/m^2^. Aligning stability was observed at 200 °C and UV irradiation of 20 J/cm^2^ for LC cells made using the PPHE100 film. Therefore, it was found that biphenyl, one of the LC precursors, modified polystyrene derivatives and can produce a next-generation vertical LC alignment system.

## 1. Introduction

The physicochemical properties—such as thermal conductivity [1,2,3,4], mechanical properties [5,6,7], and wettability [8,9]—of anisotropic molecules are influenced by their molecular alignments. For example, the thermal conductivity of anisotropic molecules could be changed by adjusting the molecular orientation in the polymer chain. The polymer chains oriented parallel with regard to the direction of heat transfer are preferred for enhancing thermal conductivity because carbon–carbon covalent bonds transport atomic thermal vibrational energy compared to van der Waals interactions between polymer interchains [1,2]. The thermal conductivity of polyethylene in parallel with regard to the polymer chain is considerably larger than polyethylene which is perpendicular with regard to the polymer chain, due to the aforementioned mechanism [10]. The mechanical properties are also associated with the alignment of anisotropic molecules. The tensile strength of materials containing anisotropic molecules oriented in parallel to the applied load is larger than that containing anisotropic molecules with a perpendicular orientation with respect to the applied load. For instance, polymer reinforced with glass fiber exhibits interesting mechanical properties according to orientation of the glass fiber. The maximum tensile strength of the polymer samples was observed for glass fiber oriented in parallel with respect to the external stress [5,6,7]. In addition, the surface properties of polymeric materials could be controlled by the implementation of anisotropic characteristics onto the polymeric surface. For example, polydimethylsiloxane (PDMS) is restricted to biomedical applications such as biosensors and implants, owing to the hydrophobic characteristics in biomedical systems [11,12,13,14], despite innumerable advantages, such as low cost and good optical transparency [15,16,17,18,19]. The surface properties of PDMS could be reformed by orienting the anisotropic molecules, such as anisotropic surfactant, vertically onto the polymeric surface. The hydrophobic parts of the anisotropic surfactant are adsorbed onto surface of the PDMS while the hydrophilic parts face out into the aqueous solution, thereby varying the surface properties of the PDMS [8,9].

Liquid crystal (LC) molecules are competitive materials with an intermediate phase between liquids and solids. The LC molecules, which are anisotropic attractive molecules, play an important role in material science when it comes to researching the relationship between the chemical structure of molecules and physicochemical properties. LC molecules can be introduced in various applications owing to their interesting physicochemical properties, such as optical and dielectric anisotropy [20]. The orientation technology of LC molecules has been developed for various applications. For instance, the vertical alignment of LC molecules, wherein the direction of the LC molecules is oriented vertically relative to the surface, has been investigated for optical sensor applications owing to susceptibility to small perturbations [21,22,23,24]. It has been found that LC molecules could be oriented by the anisotropic properties of a surface via contact and non-contact methods, such as rubbing, lithography, stretching, polarized ultraviolet irradiation, and ion beam treatment [25,26,27,28,29,30,31,32]. Among these, the rubbing technique is the conventional contact method used to align LC molecules because of its simplicity and rapidity [33,34]. Polyimide films have been commonly employed as LC alignment layers using the rubbing technique to provide considerable aligning stability in the LC alignment behavior [35,36]. However, baking processes are needed to finalize polyimide alignment layers, and the traditional baking temperature of normal polyimide films is generally over 180 °C, which is too high for the fabrication of flexible organic-based products [37,38]. Unexpected problems have been observed after the rubbing treatment, such as dust generation by electrostatic charge and physical damage on the surface of the alignment layer [39,40,41]. Non-contact methods for the alignment of LC molecules have been suggested in order to overcome weakness of the rubbing technique. Photoalignment technology has been investigated as a promising non-contact method for next-generation applications, such as flexible organic-based displays, because of the attractive advantages of photoalignment—including cleanliness, lack of limitations to surface morphology, and adaptability for large-area applications. Numerous polymers having a variety of photoreactive groups have been suggested as photoalignment layers [42,43,44]. It is widely known that polystyrene (PS) is used for many industries in a variety of forms because PS is a glassy, amorphous polymer that has clarity, flexibility, and processability [45,46]. PS films inducing planar LC alignment can be made at low temperatures suitable for the fabrication of flexible organic-based displays. It is accepted that the aligning ability of LC cells made from PS films is not good enough to make reproducible LC alignment layers; the planar LC alignment of these assembled cells cannot be maintained for more than several days. Vertical LC alignment layers made from PS derivatives via a simple polymer modification reaction have been developed, owing to advantages such as low temperature processing and good optical transparency. PS derivatives modified with phenolic compounds have been developed in order to align LC molecules vertically onto substrates using non-contact methods, because the phenolic compounds can be modified with surfaces of substrates including polymers and metals by various reactions [47,48,49,50]. For instance, the vertical alignment of LC molecules in LC cells made from PS derivatives modified with biorenewable resources, including phenolic compounds—such as capsaicin, eugenol, and vanillin—was observed. This is due to the bulky groups of the biorenewable resources which are closely related to low surface energy value owing to the steric effect of bulky groups onto the polymer surface [51,52,53,54,55,56,57]. In addition, the LC cells made using the PS derivatives containing LC precursors in the polymer—such as 4-(*trans*-4-ethylcyclohexyl)phenol [58], ethyl-*p*-hydroxybenzoate [59], and 4-ethyloxyphenol [60]—showed the vertical alignment of LC molecules. It is suggested that the similarity of the chemical structure between the LC molecules and alignment layer can induce the vertical alignment of LC molecules. The surface energy value of polymer films and the molecular alignment in the polymer chains are crucial factors in producing vertical LC alignment behavior due to the interactions and/or steric repulsions between polymer surfaces and LC molecules [61,62].

In this article, a series of phenylphenoxymethyl-substituted polystyrene (PPHE#) derivatives having an aromatic structure in the biphenyl-based moiety was synthesized to investigate LC behaviors on the alignment film made from PS derivatives structurally similar to LC molecules. This study suggests that the structural similarity between the LC molecules and alignment layer can be favorable in the vertical alignment of LC molecules. We could vary the molar content of the phenylphenoxymethyl group, and the effect of biphenyl moiety, a representative of an LC building block, in the side chain on their LC alignment behaviors could be studied very systematically. Surface characterization, such as surface energy measurement, was carried out in order to investigate the effect of the wettability on the LC alignment properties of the polymer film. The synthesis and characterization of these polymer series and the optical properties of the fabricated LC cells made from polymer films were included.

## 2. Materials and Methods

### 2.1. Materials

4-Chloromethylstyrene (90%), 4-phenylphenol (97%), and 4′-pentyl-4-biphenylcarbonitrile (5CB, 98%) (*n_e_* = 1.7074, *n_o_* = 1.5343, and Δ*ε* = 14.5, where *n_e_*, *n_o_*, and Δ*ε* indicate extraordinary refractive indexes, ordinary refractive indexes, and dielectric anisotropy, respectively) were provided from Merck Co. (Seoul, Korea). Potassium carbonate, 2,2′-azobisisobutyronitrile (AIBN), tetrahydrofuran (THF, 99%), molecular sieve (4 Å), and *N,N*′-dimethylacetamide (DMAc, 99.5%) were supplied by Daejung Co. (Busan, Korea). Methanol (99.5%) was acquired from SK Chemical Co. (Ulsan, Korea). DMAc and methanol were dried with molecular sieves (4 Å). THF was dried through refluxing with benzophenone and sodium, followed by distillation in order to remove the water. 2,2′-azobisisobutyronitrile (AIBN) was purified through crystallization using methanol. 4-Chloromethylstyrene was purified through column chromatography on silica gel, eluting with hexane to remove any residue such as *tert*-butylcatechol and nitroparaffin. Poly(4-chloromethylstyrene) (PCMS of *M*_n_ = 28,000, *M*_w_/*M*_n_ = 2.57) was synthesized using a free radical polymerization of 4-chloromethylstyrene (98.3 mmol, 15 g) with initiator, AIBN (1.0 wt % based on monomer) in THF (60 mL) under a nitrogen atmosphere at 60 °C for 24 h. After the mixture in solution was cooled at room temperature, the mixture in solution was poured into methanol to obtain a precipitate. In order to eliminate the any residue such as 4-chloromethylstyrene and low molecular weight PCMS, the precipitate was purified by a Soxhlet extraction with methanol. All other materials were used as received.

### 2.2. Synthesis of Phenylphenoxymethyl-Substituted Polystyrene

PPHE#, phenylphenoxymethyl-substituted polystyrenes—where # is the molar fraction of the phenylphenoxymethyl side groups in the polymers—were synthesized by the procedure described below. As an example, the synthesis of the phenylphenoxymethyl-substituted polystyrene PPHE100 is presented. Poly(4-chloromethylstyrene) (PCMS, 1.97 milliequivalents of repeating units, 0.30 g) and 4-phenylphenol (150 mol % compared with PCMS, 2.95 mmol, 0.50 g) were dissolved in the *N,N*′-dimethylacetamide (DMAc, 50 mL). Potassium carbonate (3.54 mmol, 0.611 g) was added to the 4-phenylphenol and PCMS mixture and then magnetically stirred at 70 °C for 24 h in a nitrogen atmosphere. In order to obtain a precipitate, the synthesized mixture in solution was poured into methanol after the mixture in solution was cooled at room temperature. The precipitate was filtered out and washed using a Soxhlet extractor with hot methanol in order to eliminate potassium carbonate and any residue. The PPHE100 obtained yields above 80% after drying overnight under vacuum. The degree (%) of substitution from PCMS to PPHE100 was interpreted by proton nuclear magnetic resonance (^1^H NMR).

PPHE100 ^1^H NMR (400 MHz, CDCl_3_, *δ*/ppm): *δ* = 1.1–2.0 (–*CH*–*CH_2_*–, 3H), 4.6–5.2 (–O–*CH_2_*–Ph–, 2H), 6.3–7.7 (*PhH*–*PhH*–O–CH_2_–*PhH*–, 13H).

Other polystyrene derivatives containing phenylphenoxymethyl side groups were synthesized by an analogous procedure used to prepare PPHE100 except for changing amounts of 4-phenylphenol in the reaction. For example, PPHE75, PPHE50, PPHE25, PPHE15, and PPHE5 were prepared with 4-phenylphenol of 0.25 g (1.48 mmol), 0.17 g (0.98 mmol), 0.083 g (0.49 mmol), 0.051 g (0.29 mmol), and 0.017 g (0.10 mmol), respectively, with slightly larger amounts of potassium carbonate (120 mol % compared with 4-phenylphenol). ^1^H NMR assignments of the respective peaks of the polystyrene derivatives (PPHE75, PPHE50, PPHE25, PPHE15, and PPHE5) are as follows:

PPHE75 ^1^H NMR (400 MHz, CDCl_3_, *δ*/ppm): *δ* = 1.1–2.0 (Cl–CH_2_–Ph–*CH*–*CH_2_*–, –O– CH_2_–Ph–*CH*–*CH_2_*–, 6H), 4.0–4.5 (Cl–*CH_2_*–Ph–, 2H), 4.5–5.0 (–O–*CH_2_*–Ph–, 2H), 6.0–7.5 (Cl–CH_2_–*PhH*–, *PhH*–*PhH*–O–CH_2_–*PhH*–, 17H).

PPHE50 ^1^H NMR (400 MHz, CDCl_3_, *δ*/ppm): *δ* = 1.1–1.9 Cl–CH_2_–Ph–*CH*–*CH_2_*–, –O– CH_2_–Ph–*CH*–*CH_2_*–, 6H), 4.2–4.6 (Cl–*CH_2_*–Ph–, 2H), 4.7–5.1 (–O–*CH_2_*–Ph–, 2H), 6.1–7.6 (Cl–CH_2_–*PhH*–, *PhH*–*PhH*–O–CH_2_–*PhH*–, 17H).

PPHE25 ^1^H NMR (400 MHz, CDCl_3_, *δ*/ppm): *δ* = 1.1–1.9 Cl–CH_2_–Ph–*CH*–*CH_2_*–, –O– CH_2_–Ph–*CH*–*CH_2_*–, 6H), 4.0–4.6 (Cl–*CH_2_*–Ph–, 2H), 4.7–5.1 (–O–*CH_2_*–Ph–, 2H), 6.3–7.7 (Cl–CH_2_–*PhH*–, *PhH*–*PhH*–O–CH_2_–*PhH*–, 17H).

PPHE15 ^1^H NMR (400 MHz, CDCl_3_, *δ*/ppm): *δ* = 1.0–1.8 Cl–CH_2_–Ph–*CH*–*CH_2_*–, –O– CH_2_–Ph–*CH*–*CH_2_*–, 6H), 4.1–4.7 (Cl–*CH_2_*–Ph–, 2H), 4.7–5.2 (–O–*CH_2_*–Ph–, 2H), 6.0–7.6 (Cl–CH_2_–*PhH*–, *PhH*–*PhH*–O–CH_2_–*PhH*–, 17H).

PPHE5 ^1^H NMR (400 MHz, CDCl_3_, *δ*/ppm): *δ* = 1.2–2.0 Cl–CH_2_–Ph–*CH*–*CH_2_*–, –O– CH_2_–Ph–*CH*–*CH_2_*–, 6H), 4.4–4.8 (Cl–*CH_2_*–Ph–, 2H), 4.8–5.1 (–O–*CH_2_*–Ph–, 2H), 6.0–7.3 (Cl–CH_2_–*PhH*–, *PhH*–*PhH*–O–CH_2_–*PhH*–, 17H).

### 2.3. Film Preparation and LC Cell Assembly

Each solution of the PPHE# in tetrahydrofuran (1 wt %) was filtered by a poly(tetrafluoroethylene) (PTFE) membrane that has a pore size of 0.45 μm. Thin polymer films were made through spin-coating (2000 rpm and 60 s) onto 2.0 × 2.5 cm^2^ glass substrates using about 3.5 mL of 1 wt % THF solution of PPHE5, PPHE15, PPHE25, PPHE50, PPHE75, and PPHE100. The LC cells were made by assembling the prepared films using spacers that have a thickness of 4.25 μm. We selected 4′-pentyl-4-biphenylcarbonitrile (5CB) in fabricated LC cells to analyze the correlation between the alignment layer and LC molecules by physicochemical interaction because of the physicochemical properties of 5CB—such as its accessible nematic range near room temperature, good chemical stability, and high positive dielectric anisotropy, as reported in previous studies [63,64,65,66]. The fabricated cells were filled with nematic LCs (5CB) and were then sealed using epoxy glue.

### 2.4. Instrumentation

The proton nuclear magnetic resonance (^1^H NMR) measurements were collected on Agilent MR400 DD2 (Agilent Technologies, Inc., Santa Clara, CA, USA) NMR spectrometer at a sample concentration of 20 mM deuterated solution. The average molecular weight (*M*_n_) and polydispersity index (*M*_w_/*M*_n_) of the synthesized polymer was measured by Waters gel permeation chromatography (GPC) with respect to monodisperse polystyrene standards using tetrahydrofuran as the eluent and a differential refractometer as the detector. A Q-10 (TA Instruments, Inc., New Castle, DE, USA) equipped with RCS40 (TA Instruments, Inc., New Castle, DE, USA) was employed to analyze thermal properties of the polymer by differential scanning calorimetry (DSC) at a heating rate of 10 °C/min and a cooling rate of 100 °C/min under a nitrogen atmosphere. The glass transition temperature (*T*_g_) was detected in the second heating run over the range −40 to 200 °C. The contact angles were measured right away using a Krüss DSA10 (KRÜSS Scientific Instruments Inc., Hamburg, Germany) contact angle analyzer fitted with drop shape analysis software after deposing the water and diiodomethane droplets on the polymer films. The average volume of the droplets used for the contact angle measurement was 5 μL. The contact angles for each sample were determined four or more times on three independently prepared polymer films. The representative values were averaged. A Nikon Eclipse E600 POL (NIKON, Inc., Tokyo, Japan) fitted with a polarizer and Nikon, Coolpix 995 digital camera (NIKON Inc., Tokyo, Japan) was applied to take polarized optical microscopy (POM) images for the LC cell. In order to confirm the reliability to apply harsh environment, the ultraviolet (UV) stability of the LC cells was confirmed using a VL-6.LC lamp (λ_max_ = 365 nm, Vilber Lourmat, Paris, France). A UV detector, GT-513 (Giltron, Seoul, Korea) was used to measure the intensity of irradiated UV light on the LC cells during UV light irradiation. The multi hotplate stirrer (DAIHAN Scientific Co., Ltd., Wonju, Korea) was used as a heating element to investigate the thermal stability of the LC cells. The LC cells were heated from room temperature to 100, 150, and 200 °C. The surface energy values were calculated by Owens–Wendt equation

(1) $$\gamma_{sl}=\gamma_{s}+\gamma_{l}-2{\left(\gamma_{s}{}^{d}\gamma_{l}{}^{d}\right)}^{1/2}-2{\left(\gamma_{s}{}^{p}\gamma_{l}{}^{p}\right)}^{1/2}$$

where, *γ_l_* is the surface energy of the liquid, *γ_sl_* is the interfacial energy of the solid/liquid interface, *γ_s_* is the surface energy of the solid, *γ_l_^d^* and *γ_l_^p^* are known for the test liquids, and *γ_s_^d^* and *γ_s_^p^* can be calculated from the measured static contact angles [67].

## 3. Results and Discussion

**Figure 1:**
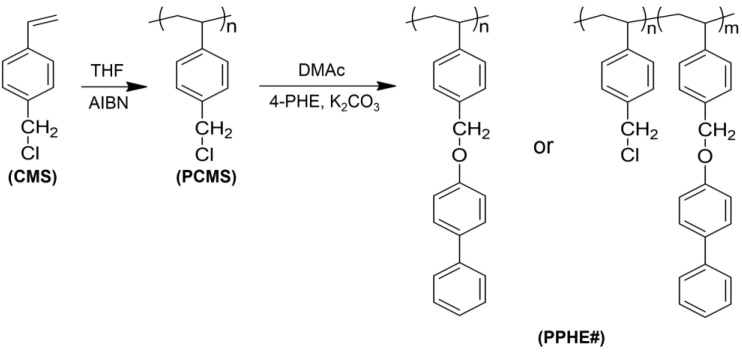
Synthetic route of phenylphenoxymethyl-substituted polystyrene films (PPHE#), where # indicates the mole percent of 4-phenylphenol containing monomeric units in the polymer.

**Figure 2:**
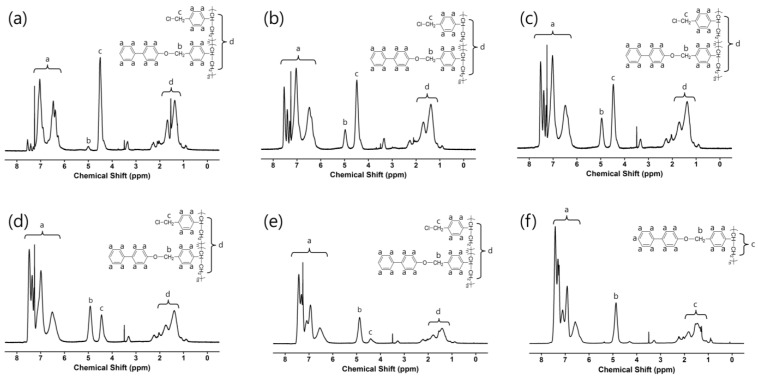
Proton nuclear magnetic resonance (^1^H NMR) spectrum of (**a**) PPHE5, (**b**) PPHE15, (**c**) PPHE25, (**d**) PPHE50, (**e**) PPHE75, and (**f**) PPHE100.

Figure 1 shows the route of synthesis for the phenylphenoxymethyl-substituted polystyrene, PPHE# (homopolymer PPHE100 and copolymers PPHE75, PPHE50, PPHE25, PPHE15, and PPHE5), where # is the molar content (%) of phenylphenoxymethyl side groups. As previously reported, the degrees of substitution reaction of the chlorine in poly(chloromethylstyrene) (PCMS) by nucleophilic compounds including the phenolate compounds, were observed to almost agree with feeding amount of the substitution [68,69]. The acidity of phenolic compounds was affected by the electron-withdrawing substituents group to the hydroxyl group [70,71]. The phenol group in the 4-phenylphenol can be easily dissociated to the phenolate and the proton, which is a strong nucleophile, since the structure of the phenolate anion could be stabilized by resonance structures. Furthermore, the benzylic carbon in the PCMS is relatively electron-deficient, due to the electron-withdrawing groups, *viz.* the chlorine and phenyl groups, which are attached directly to the carbon [72]. In addition, the structure of the transition state in the substitution reaction could be stabilized by conjugation with the benzene ring. Hence, the high conversion rate in the polymer modification reaction could be demonstrated by the electrophilicity of benzylic carbon in PCMS and the chemical structure stability of the phenolate anion as a nucleophile. The copolymers—PPHE5, PPHE15, PPHE25, PPHE50, and PPHE75—with different degree-of-substitution ratios (%) were obtained by varying the 4-phenylphenol amounts in the reaction. Conversions from chloromethyl to phenylphenoxymethyl group are almost 100% when 150 mol % of phenylphenoxymethyl was used at 70 °C for 24 h, as determined in the assignment of the corresponding proton peaks of the phenylphenoxymethyl-substituted polystyrene PPHE100. The mole percent of phenylphenoxymethyl containing monomeric units in the obtained polymers was demonstrated by proton nuclear magnetic resonance (^1^H NMR) measurement. The ^1^H NMR spectra and assignments of the respective peaks of the polymers—such as PPHE100, PPHE75, PPHE50, PPHE25, PPHE15, and PPHE5—can be seen from Figure 2. The ^1^H NMR spectrum of PPHE100 (Figure 2f) is explained as an example. The ^1^H NMR spectrum of PPHE100 indicated the presence of protons of the phenyl ring in the styrene backbone and the proton peaks from the phenyl ring in the phenylphenoxymethyl side chains (*δ* = 6.3–7.7 ppm (peak a)). In addition, the proton peaks from alkyl groups of oxymethyl and styrene backbone were identified at (*δ* = 4.6–5.2 ppm (peak b) and *δ* = 1.1–2.0 ppm (peak c)). The degree of substitution from chloromethyl to phenylphenoxymethyl was calculated to be almost 100% by comparing the integral ratio of the phenylphenoxymethyl peaks at 6.3–7.7 ppm and the backbone peaks at 1.1–2.0 ppm. Similar integrations and calculations for PPHE5, PPHE15, PPHE25, PPHE50, and PPHE75 were performed, and results were generally within ±10% of the expected values of synthesis. These prepared polymers showed good solubility in medium-polarity solvents that have low boiling points, including THF and chloroform. All samples exhibited a good solubility in various solvents for the application of PPHE# as thin-film materials. We selected THF as the coating solvent to make thin-film materials, because of its low toxicity and good biodegradability [73]. These polymer films can be fabricated using a wet process at a low temperature for next-generation applications.

**Figure 3:**
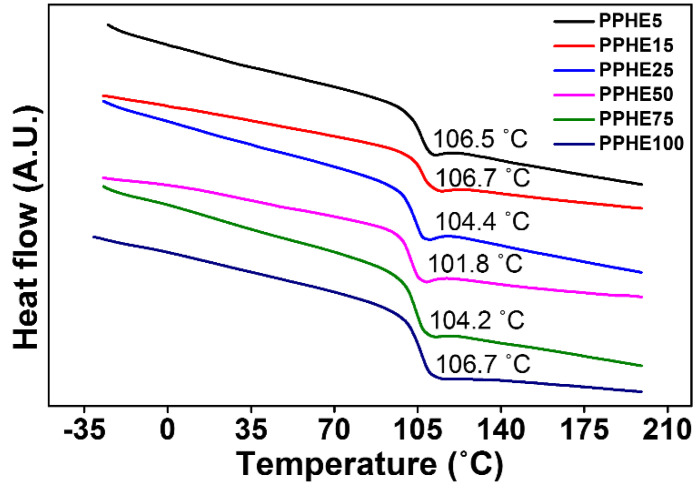
Differential scanning calorimetry (DSC) thermogram of PPHE#.

The thermal properties of these polymers, PPHE#, were studied using differential scanning calorimetry (DSC), and all polymers exhibited amorphous behavior, because only the glass transition was found from their DSC thermogram. The *T*_g_ values of polystyrene derivatives slightly increased or decreased according to the different molar content of the side group as shown in Figure 3. As described in the previous studies, the *T*_g_ of the polymers was affected by two main factors—chain flexibility and intermolecular interaction [74]. The changes in *T*_g_ of polymer were determined by interplay between these two effects. For example, the decrease in *T*_g_ values of the PS derivatives with increasing molar content of the bulky phenylphenoxymethyl side chains in the polymer was ascribed to the increase in steric volume in the polymer, as previously reported [75,76,77], while the increase in the *T*_g_ values of the PS derivative was attributed to an increase in the *π-π* and van der Waals interactions among the phenylphenoxymethyl side chains [75,76,78,79]. The *T*_g_ values of PPHE5, PPHE15, PPHE25, PPHE50, PPHE75, and PPHE100 are 108.1, 109.6, 106.9, 104.9, 105.0, and 106.7 °C. In conclusion, the *T*_g_ value of the PS derivatives is determined by a play-off between the free volume effect and the intermolecular interaction effect of the polymer chain. It was found that the *T*_g_ values of these polymers, PPHE#s, were higher than 100 °C due to their aromatic structure in the biphenyl-based PHE moiety. This result suggests that polystyrene substituted with phenylphenoxymethyl (PPHE#) containing the aromatic biphenyl moiety—a representative precursor in LC molecules—has a thermal stability which is higher than that of the previously reported PS derivatives containing alkyl aromatic LC precursors in the polymer—such as 4-(*trans*-4-ethylcyclohexyl)phenol [58], ethyl-*p*-hydroxybenzoate [59], and 4-ethyloxyphenol [60].

**Figure 4:**
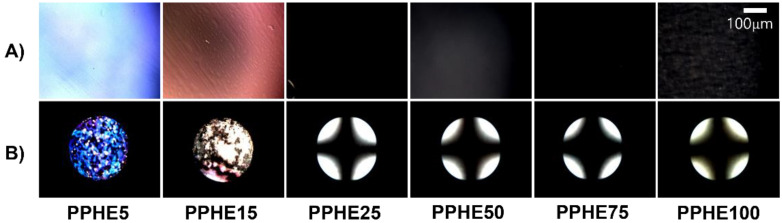
(**A**) Orthoscopic, (**B**) conoscopic polarized optical microscopy (POM) images of the LC cells made using PPHE# (PPHE5, PPHE15, PPHE25, PPHE50, PPHE75, and PPHE100) films.

It is widely known that the LC alignment behavior can be affected by the interactions at the interface between alignment layer and LC molecules [80,81,82]. Therefore, LC cells made using films of phenylphenoxymethyl-substituted polystyrene derivatives structurally similar to LC molecules were fabricated using 5CB to study the LC alignment behavior of the polymer films. The LC cells fabricated from PPHP# films with a phenylphenoxymethyl side group content of less than 15 mol % (PPHP5 and PPHP15) showed planar LC alignment behavior, while good uniformity of vertical LC alignment behavior was observed for LC cells fabricated with the polymer films with a phenylphenoxymethyl side group content of at least 25 mol % (PPHP25, PPHP50, PPHP75, and PPHP100). We investigated the LC alignment performance on PPHE# films by observing their polarized optical microscopy (POM) images, as can be seen from Figure 4. A random planar LC alignment performance was observed for the LC cells made using the PCMS film (figure not shown). As the molar content of the phenylphenoxymethyl containing a monomeric unit in the PPHE# was 5 and 15 mol %, the LC cells fabricated with the PPHE# film exhibited random planar LC alignment in the conoscopic POM images. In contrast, at a molar content of 25–100 mol %, the LC cells fabricated with the PPHE# film exhibited a uniform vertical LC alignment, and it can be confirmed by the Maltese cross pattern of the conoscopic POM images. Based on the LC alignment results, we observed a general tendency that the polymers having higher molar content of phenylphenoxymethyl side groups with a similar molecular structure prefer vertical LC alignment.

**Figure 5:**
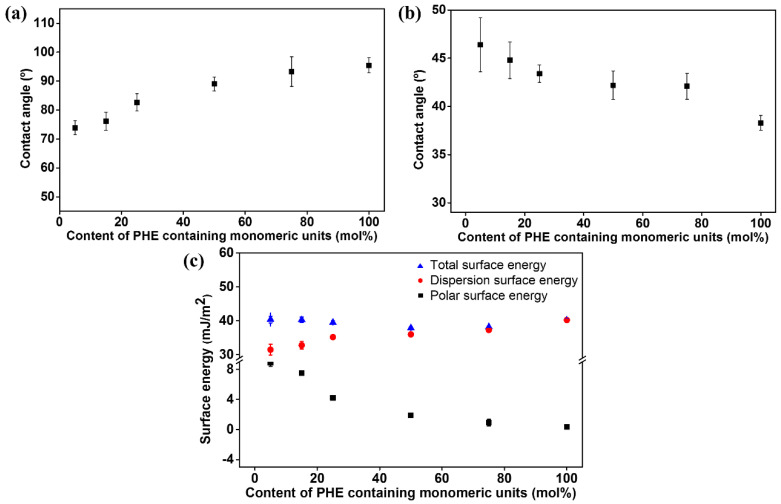
(**a**) Water, (**b**) diiodomethane contact angle, and (**c**) surface energy values of PPHE# films according to the molar content of the phenylphenoxymethyl moiety in the side group.

It is widely known that the vertical alignment behavior of the LC molecules on the alignment layer could be ascribed to the surface energy of the alignment layer which is smaller than the surface tension of the LC [83,84,85,86]. Therefore, we demonstrated the LC alignment behaviors of the PPHE# films using surface energy measurements, one of the surface characterization techniques. Figure 5 and Table 1 show the surface energy values of the PPHE# films calculated from the static contact angles for water and diiodomethane on these polymer films. The total surface energy values are the summation of the polar and dispersion contributions and were calculated from the Owens–Wendt equation. The total surface energy values of PPHE5, PPHE15, PPHE25, PPHE50, PPHE75, and PPHE100 are 40.4, 40.3, 39.4, 37.8, 38.2, and 40.5 mJ/m^2^, respectively. The dispersion surface energy values of PPHE# according to the molar content of the phenylphenoxymethyl moiety increased, to 31.5, 32.8, 35.2, 36.0, 37.3, and 40.2 mJ/m^2^. The polar surface energy values of PPHE# decreased to 8.9, 7.5, 4.2, 1.8, 0.9, and 0.3 mJ/m^2^ with increasing the molar content of the phenylphenoxymethyl moiety in the side groups. We found that the vertical LC alignment performance correlates well with the polar surface energy of the PPHE# films. As shown in several studies, the polar surface energy of the alignment layer can affect the LC alignment performances [86,87,88]. Therefore, it is suggested that the vertical LC alignment behavior is affected by the polar surface energy of the polymer films which is lesser than approximately 4.2 mJ/m^2^ due to the unique structure of the phenylphenoxymethyl side chain.

**Figure 6:**
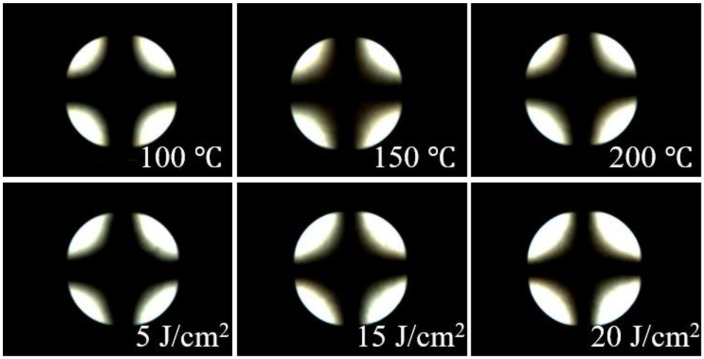
Conoscopic POM images of the LC cells made using PPHE100 films, after thermal treatment at 100, 150, and 200 °C for 10 min and ultraviolet (UV) treatment at 5, 15, and 20 J/cm^2^, respectively.

In order to confirm the reliability of LC cells composed of the polymer films, a stability test of the LC alignment performance was carried out under the harsh conditions, viz. high temperature and ultraviolet (UV) exposure. The thermal and UV stabilities of the LC cells made from the PPHE100 films were demonstrated from the POM image after heating and ultraviolet irradiation (Figure 6). In the case of the thermal stability test, the LC cells fabricated with PPHE100 films were heated using the hot plate for 10 min under different temperatures at room temperature, 100, 150, and 200 °C, respectively. Furthermore, in order to confirm the UV stability of LC cells fabricated with PPHE100 films, the LC cells were irradiated with UV rays at 5, 15, and 20 J/cm^2^, respectively. As shown in Figure 6, no distinguishable difference in the vertical LC alignment of the LC cells prepared from PPHE100 films could be observed from the conoscopic POM images, indicating that the vertical LC aligning ability was maintained when heated at 200 °C for 10 min and irradiated with exposure energy of 20 J/cm^2^. Therefore, PPHE100 LC cell can be applied to high temperature and UV irradiation conditions. It is important to align the anisotropic materials in one direction, such as the liquid crystal elastomers (LCEs) and photocurable materials in LC composite system, as well as cyanobiphenyl-based LC which is one of the small molecule LCs. We believe that comb-like polystyrene derivatives having low processing temperature and good optical transparency and high solubility in common organic solvent can be a suitable candidate of the alignment layer for future applications in biomedical, LC laser, and 4D printing using LCs [89,90,91,92].

## 4. Conclusions

A copolymer series of polystyrene substituted with phenylphenoxymethyl (PPHE#) containing the biphenyl moiety, a representative precursor in LC molecules, was synthesized in order to evaluate the LC alignment behavior of the polymer films. Interestingly, the LC cells made from the films of phenylphenoxymethyl-substituted polystyrene having only an additional phenylphenoxymethyl side group showed vertical LC alignment behavior. The vertical LC aligning ability was observed for the LC cells made using the polymer films with a higher molar content of phenylphenoxymethyl side groups. The LC cells made from polymer films having 25 mol % or more of phenylphenoxymethyl (PPHE25, PPHE50, PPHE75, and PPHE100) side groups showed vertical LC aligning ability, while the LC cells—made from PPHE# films having less than 25 mol % of phenylphenoxymethyl side groups—exhibited random planar LC alignment. These results suggest that the LC precursor structures in the polymer side chains can be advantageous in the vertical alignment of LC molecules. The vertical LC alignment behavior was correlated with polymer films that have polar surface energies lower than 4.2 mJ/m^2^, due to the unique structure of the phenylphenoxymethyl side chain. Therefore, phenylphenoxymethyl-substituted polystyrenes could be promising candidates for the LC alignment layer for next-generation applications using wet processes at low temperatures. This work can provide the basic information for the design of the LC alignment layer using LC precursors containing polymer films.

## Figures and Tables

**Table 1:** Surface energy values and LC alignment properties.

Polymer Designation	Contact Angle *^a^* (°)		Surface Energy *^b^* (mJ/m^2^)			Vertical LC Aligning Ability
	Water	Diiodomethane	Polar	Dispersion	Total	
PPHE5	73.8 (2.4) *^c^*	46.4 (2.8) *^c^*	8.9 (0.5)*^c^*	31.5 (1.6) *^c^*	40.4 (1.0) *^c^*	No
PPHE15	76.1 (3.1)	44.8 (1.9)	7.5 (0.3)	32.8 (1.1)	40.3 (0.8)	No
PPHE25	82.6 (3.0)	43.4 (0.9)	4.2 (0.1)	35.2 (0.6)	39.4 (0.5)	Yes
PPHE50	89.0 (2.4)	42.2 (0.5)	1.8 (0.3)	36.0 (0.1)	37.8 (0.4)	Yes
PPHE75	93.3 (5.1)	42.1 (0.3)	0.9 (0.4)	37.3 (0.3)	38.2 (0.1)	Yes
PPHE100	95.5 (2.6)	38.3 (0.8)	0.3 (0.2)	40.2 (0.2)	40.5 (0.1)	Yes

*^a^* Measured from static contact angles. *^b^* Calculated from Owens–Wendt equation. *^c^* Standard deviations were inserted in parentheses.

## Data Availability

The data presented in this study are available on request from the corresponding author.

## Abbreviations

AIBN	2,2′-Azobisisobutyronitrile
5CB	4′-Pentyl-4-biphenylcarbonitrile
DMAc	*N,N*′-Dimethylacetamide
DSC	Differential scanning calorimetry
^1^H NMR	Proton nuclear magnetic resonance
*T*_g_	Glass transition temperature
THF	Tetrahydrofuran
LC	Liquid crystal
PCMS	Poly(4-chloromethylstyrene)
PPHE#	Phenylphenoxymethyl-substituted polystyrene
POM	Polarized optical microscopy
PS	Polystyrene
